# Extra Supplement—The Nursing Mirror

**Published:** 1889-07-13

**Authors:** 


*The Hospital,*
July 13, 1889.

[*Extra Supplemen*

### The Nursing Mirror.

All Contributions for this Supplement should be addressed "The Nursing Mirror," care of the Editor, 139-140, Salisbury Court, Fleet Street, London, E.C.

### En Passant.

THE TEACHING OF NURSES.—We are glad to be able to commence this week a series of articles on physiology, which, we hope, will help nurses preparing for examinations. The articles are made as interesting as possible, as the writer (a certificated sister) believes that dry facts are less easily remembered than amusing illustrations. We hope that all nurses, whether they are preparing for examinations or not, will read and enjoy these papers, and find something in each to learn and remember.

CHURCH ARMY NURSES.—We learn, with pleasure that the Church Army is about to widen its nursing ranks. Ladies are to be taken into the home in Edgware-road, and trained in the combined duties of tending suffering bodies and souls. The Army will do well to train its nurses more thoroughly if it contemplates sending them to India; and those who are chosen for the special mission to lepers should be taught some facts concerning that disease. The nurses have done good work in London: their bright faces beneath the black bonnets (on which the silver cross shines forth as a token) have carried hope to many a dreary home, and we trust that their labours in a larger field will be equally blessed.

SALISBURY.—There is a delightful old infirmary at Salisbury, a huge red-brick structure, with a garden running down to the river. Many nurses are trained here for institutions and sisterhoods, and paying probationers are also taken. The nursing arrangements are excellent, under the able superintendence of Miss Morris, and a certificate is given at the end of a year. There is a home in connection with the hospital, from which private nurses are sent out. These nurses have a neat uniform with a distinctive violet veil; the infirmary nurses have no outdoor uniform. There is also a large provident dispensary at Salisbury, to which a nurse is attached. Fisherton House Asylum in the neighbourhood is at present in want of a matron. Salisbury was very gay last week; the Duchess of Albany opened a bazaar in the Palace grounds, and the assizes also commenced, and brought many strangers to the town. A nurse who secures a post in this delightful old cathedral city may consider herself remarkably lucky.

SUMMER AFTERNOONS.—Anything to do with water is agreeable during the heat of summer, so we have two suggestions to make to our readers this week. First, we would recall to mind the penny steamers which run up and down the Thames. Except about 5 p.m., when gentlemen are returning from business, these steamers are not crowded, and a trip up to Chelsea and back is a pleasant and cheap way to spend an hour. But these steamers are so well known to nurses we need not say much about them, but swimming-baths offer a pleasure of which few nurses take advantage. The Paddington Baths, close to Queen's-road Station, or the Royal Oak 'busses, are excellent, and admission is only eightpence. St. George's Baths, in Buckingham Palace-road, are only open to ladies on Wednesday mornings. The St. Marylebone Baths, in the Marylebone-road, are open daily to women. The St. Pancras Baths are open on Saturdays at King-street, and on Wednesdays at Tottenham-court-road. Next to sea-bathing there is nothing more refreshing than a swim in a good large bath; and if any nurse cannot swim the sooner she learns the better.

FLORENCE NIGHTINGALE. — THE HOSPITAL for July 27 will contain a biography of Miss Nightingale, and the monthly part for August will contain an excellent portrait and autograph of the head of the nursing world. The portrait will be obtainable with the weekly parts if an extra 3d. is paid.

COOKING FOR INVALIDS.—The Edinburgh School of Cookery has lately devoted much attention to the special branch of sick-room requirements. A course of lectures on cookery suitable for invalids has been delivered at the Infirmary, at the Royal Hospital for Sick Children, and at the Longmore Hospital to the nurses in charge of the various wards. And, also, four lectures have been delivered to the medical students of the infirmary. Was there a twinkle in the corner of the cook's eye as she demurely instructed the young men in the mysteries of beef-tea? And did the students find it a pleasant change from the study of pharmacology and therapeutics to watch the simmering of a stewpan, and to whip the whites of eggs? The report does not reply to these questions, but imagination points out that doctors learning cooking, and nurses studying pathology, is an amusing sign of the times.

INSTITUTES AT BATH.—Mr. Radway rashly stated there was no institute for trained nurses at Bath, and of course the authorities of the Homœopathic Hospital came down on him at once. Dr. Percy Wilde says: "We take our 'probationers' entirely from the educated classes, and in addition to ordinary medical and surgical training, they receive instruction in the administration of baths, the application of massage, and the use of electrical appliances. After twelve months' training they alternate private nursing with work in the ward, and the result has not been only satisfactory from an educational standpoint, but from a financial one. Practically the earnings of private nursing entirely cover the cost of the hospital nursing, and if our funds had been large enough to allow us to make our arrangements on a more extensive scale, there would have been a considerable profit from this source. This should be satisfactory to those who propose to follow the same system at the Royal United." Dr. Wilde very naturally wishes that the Homœopathic Hospital was more often remembered, especially on Hospital Saturday and Sunday.

ORKNEY.—The Orkney Isles are so remote that it is difficult to arouse English interest in them, and yet there are few towns of the size of Kirkwall so well supplied with hospital accommodation. The munificence of the late Mr. John Balfour, of Balfour and Trenabie, during his lifetime, has hitherto obviated the necessity for appeals to public generosity; and now from the same source, combined with a bequest in 1887 of £120 from the late Mrs. Ann Inkster of Eunson, still further benefits are about to be conferred on the town and county. The Balfour Trustees, finding themselves in possession of a surplus of about £500, have resolved on the erection of a new fever hospital. It is proposed to build a cottage hospital on the site of the old fever hospital. There will be three wards, containing in all fourteen beds, besides accommodation for the matron and nurses. As showing the necessity for such an undertaking, it may be mentioned that within the last month or two there have been twelve fever patients in the hospital, the present number being six. During the year 1888 the total number of patients treated in Balfour Hospital was eighty-one, of which twenty-nine were fever cases, and fifty-two ordinary patients.

### Physiology for Nurses.

### No. I. The Skin.

The skin is by no means the very simple covering to the body it appears to be to the naked eye. Its use as covering and protection to the body is its least important function; its real use is to help to keep the body pure and healthy, and so it is called an excretory organ; that is, it throws off many waste products which the body is far better without, and so helps to maintain life and health. In old days people used sometimes to be punished by varnishing the body all over, so that the waste products were kept in instead of being thrown off by the skin, and the result was that those people died a most miserable death. Long ago in Rome, when emperors used to rule over Italy, there were great festivities when one of those emperors came to the throne. A Roman mother, who had a beautiful little son of four or five years old, thought she would make him look like an angel following the Emperor's procession, so she covered him all over with gilt, and fastened gilt wings on to his shoulders, and everyone, including the Emperor, admired him very much. At night the mother thought she would like to see her angel boy in bed asleep, and she went and looked at him, and the little fellow was, indeed, an angel, for he was dead. The gilt stuff put on his skin had prevented the waste matters from escaping, and the consequence was death. You all know more or less fully the difference it makes to us if our skins are healthy or not, and what trouble and annoyance even a slight skin disease is, therefore no one can be said to have good health if their skin is not healthy and able to do its duty properly.

The skin is composed of three distinct layers. There is the outside skin, which we can see and touch, which is called the "cuticle," or scarf skin, or epidermis. It is formed of long, narrow, dry scales, piled one on the top of another. The outside ones get dryer and dryer, and are perpetually being rubbed off. Even without our knowing it we are constantly shedding a sort of very fine powder, which is these scales of the cuticle broken up. Those parts of the skin which are exposed to the air, such as the hands and face, shed the cuticle most rapidly. In scarlet fever this same skin is shed during the so-called peeling time in much larger pieces, and, therefore, they attract more notice. Immediately below this dry, scaly layer comes the mucous layer, or cutis, which looks different to the outside skin, but the differences between the two are simply due to the fact that the under is protected from the air and does not get such direct pressure as does the outside skin. You can see the differences between the two layers any day that you happen to raise a blister on your skin. If you snip a corner of the blister and let out the fluid which has probably collected, and turn part of the skin back, it will look very red beneath, and this is the mucous layer.

Further, if you took the skin away altogether, you would be surprised to find how soon the tender red skin below becomes hard and painless. This is because the cutis has become changed into cuticle. If you could take a slice of skin out of, say, your finger, and looked at it with a very strong magnifying-glass, you would find it composed of a number of more or less round clear cells piled one above the other like bricks in a wall, and these cells are kept in place by a clear thick fluid, which acts like cement. The upper layers of these cells are not as round as the lower ones, especially in those parts, like the feet and hands, where they naturally get pressed out, and as the outer skin gets worn away these scales take their place and become more and more flattened, and dry, and scaly. Another point about the upper layers of these cells is that in the centre of each are specks of colouring matter called pigment, and these give the colour to the skin. When there are many of these pigmented or coloured cells we get a dark, dusky skin. Thus, the negro's skin is dark because of all the pigment it contains; and a very fair-skinned person has less pigment. Again, an "albino," who has colourless skin, white hair, and red eyes, has no pigment cells, and never had even from birth, and no explanation can be given for its absence. Sometimes you find people whose skin has brown patches in some parts and white in others. This is because all the pigment is collected in the brown patches and is absent in the white ones. Such people also will probably have white patches of hair on their head corresponding with the white patches of skin on the scalp. In others you find moles on the skin, which may have been there from birth. These, again, are due to excessive pigment in one small spot. The pigment in the skin can be increased by heat, whether of the sun or fire, because heat draws the blood to the surface of the skin, and the blood brings the pigment to the skin.

That is why, when you have been into the country for a holiday in the summer, you may come back so brown, or may be freckled. That is the effect of the sun on your pigment cells, and in the Black Country, about Stafford and Wolverhampton, and others of those towns in the Midlands, where there are great iron works, and men have to work in the heat of great furnaces for many hours together, their faces and arms and hands get very brown. But after you have been back from the country for a little while, and if the furnacemen changed their occupation, and became, say, porters in a warehouse in a town, their skins would soon become white again; the browning effect does not last when the person is removed from the heat.

Below the mucous layer or cutis is a further layer, called the true skin, which contains the coiled ends of the sweat-glands; the other ends come straight up through the mucous layer and cuticle, and each gland has a very small opening on the surface. You cannot see this opening very distinctly with the naked eye, but if you use a magnifying-glass I think you will manage to make it out. Any of you who have read a book called "Gulliver's Travels" will remember that in one of his journeys Gulliver visits a country of giants, and he was very much shocked at the pitted appearance of their skins; but, after all, it is only what can be seen with a good microscope. These glands are about a quarter of an inch long, and in the twenty-four hours they carry off about two pounds weight of what is called invisible perspiration. It is called invisible to distinguish it from the visible perspiration when anyone is taking active exercise and gets hot. The invisible perspiration is always coming away without our knowing anything at all about it. But you can readily understand that if from any cause this invisible perspiration were checked, and these two pounds weight of impurities kept in the body instead of being allowed to escape, that it would not be good for the health, to say the least of it.

As well as these sweat-glands there are also the oil-glands, which can be easily seen on the side of the nostrils, though the fact of their being seen proves that they are not quite healthy. They contain a sort of oily matter, like butter, the use of which is to provide oil to the cuticle to keep it soft and flexible, and to the hair to nourish it. Ducks and other birds that live a great deal in the water have plenty of this oil in their feathers to keep them dry, and prevent their becoming so bedraggled as to prevent their swimming owing to the weight of water they would have to carry about with them. When their oil glands act properly they only produce just sufficient oil, and any excess is carried away, but if the skin is not quite healthy and the action of these glands is sluggish, this oily matter remains in the gland and attracts dirt to itself, and thus a black speck appears—such specks are commonly called "blackheads." The best plan is to squeeze out the oily matter which causes the speck either with the nails or with a watchkey placed over it, so that you can squeeze all round, and the oily matter comes up into the hollow part in the centre of the key. The parts where these specks appear should be well bathed with water as hot as can be comfortably borne, and then rubbed well with a rough towel so as to stimulate the glands to do their work better. These specks should not be allowed to remain or to grow large, or the part round them will become inflamed and troublesome, and a very sore spot may come which it will not be easy to get rid of again.

*(To be continued.)*

### The National Pension Fund.

### THE PRINCESS OF WALES BECOMES PRESIDENT.

A BONUS FUND OF £26,000.

With what pride and what pleasure do we announce the extraordinary success of the Pension Fund! Not only have the thousand nurses joined, but the Princess of Wales, well-known for her discrimination in only patronising associations of proved value, has become the president of the fund. Then those gentlemen who deposited £20,000 until the fund should be started have given that money to a bonus fund, and those 1,000 nurses who have been first in the field are presented with £5,000 as a bonus fund all their own, which will yield some £14,000 in reversion, to be entirely divided amongst them when their pensions commence to be paid.

In the words of the report: "As upwards of 1,000 annuity proposals have been obtained, the fund is now legally constituted. The policies issued represent a premium income of sufficient extent to make the loading—*i.e.*, the amount provided by the actuary for expenses—equal to four-fifths of the whole expenditure under every head since business was commenced in July, 1888. The remaining fifth, together with the expenses incurred between the registration of the society in February and the commencement of business in July, 1888, have been defrayed by Mr. Edward F. Coates, who has given 250 guineas, and by Messrs. C. de Murietta and Co., who have contributed 500 guineas for the special purpose. The foregoing donations enabled the manager to report that all the money contributed to the bonus fund, together with the interest derived from the deposit of £20,000 in the Court of Chancery, is now available for distribution to the nurses in bonuses without any deduction whatever. The four gentlemen who had deposited the £20,000 as security for the policyholders have now, as we have already said, given this money absolutely to the fund. Wishing to show some mark of appreciation towards those of the first 1,000 nurses who have joined the fund unaided by any institution, they have determined to give £5,000 thereof towards a special bonus fund, to enable the council to give to each of the said nurses a substantial reversionary bonus when her pension commences."

Altogether there is good cause for congratulation, for in the future no woman who devotes her life to nursing in the British Empire need suffer privation or be without adequate help in sickness or old age. The fund has great financial strength; is supported by several of our greatest City merchants; is officered by men of well-known business capacity; and last, but not least, has won the approval of the Prince and Princess of Wales. All nurses ought to be truly grateful to those who have given so munificently to this fund, and to the council and managers, who have expended so much time and thought on a scheme which will live and grow through the ages, and be a never-ending blessing to a noble band of women. Those are the true friends of nurses who help them in a practical manner; who go steadily and quietly forward with the work they undertake, and who manage to achieve substantial success for schemes which are fitted to survive and to flourish.

It has been determined to raise the General Bonus Fund at once to at least £50,000. Everybody interested in the welfare of nurses is invited to qualify as a governor of the fund by subscribing £2 2s. per annum or sending a donation of £25 (a life governor's qualification) to Mr. E. Clifford, the manager, 8, King-street, E.C.

### Everybody's Opinion.

*[Correspondence on all subjects is invited, but we cannot in any way be responsible for the opinions expressed by our correspondents. No communications can be entertained if the name and address of the correspondent is not given, or unless one side of the paper only be written on.]*

### REGISTRATION.

Miss C. J. Wood, writing to the *Evening News* and *Globe*, states: So much misapprehension is abroad concerning the legal registration of nurses, and the plan of action proposed by the British Nurses' Association, that I ask space in your journal to place the matter before your readers. The legal registration of the members of a profession is no new departure. Doctors, lawyers, soldiers, sailors, and clergymen are all registered, and many trades are now demanding the same system for themselves. It is simply this—the placing of the names of duly qualified persons on a list to be published yearly, and offered for sale. The British Nurses' Association, representing the leading medical men of the kingdom and a large portion of the nursing profession, proposes to petition the Queen in Council to grant them a Royal charter incorporating the association, and authorising it to keep such a register. The provisions of the charter must be carried out by a registration board composed of medical men and nurses; this board would certainly accept as proof of training the certificates now given by the recognised training schools of nurses, together with testimonials of good character, and on submitting these the nurse's name would be placed on the register. The responsibility of deciding upon the qualification of a nurse would therefore really rest with the training school, which could withhold the certificate, without which the nurse could not register. There is not the slightest interference here between the training schools and their pupils, no attempt at reducing the art of nursing to a "dead level," no doing away with the tests of personal fitness to which every hospital subjects its practitioners. The ubiquitous individual who, under various signatures, is making all these false statements, is merely attempting to blind the public to the fact that at present there is no protection against any woman, however destitute of knowledge or character, choosing to call herself a trained nurse, and no means of preventing trained nurses who disgrace their calling once and again from continuing to do so. Stress need not be laid upon the dangers to which the public are exposed at the hands of both classes. Another misstatement I should like to correct. The Hospitals Association did not originate the scheme of registration; it sprang out of a letter from myself written to that association about four years ago, and as the result of the suggestions contained in that letter a conference of the leading matrons in the metropolis was called; out of this we formed a sub-committee to work out the idea. Then we learned that a mixed lay and professional body is not the best for working out strictly professional schemes, and that registrations under a voluntary association is practically valueless; and so we seceded and formed the British Nurses' Association. I have the correspondence in my possession to prove the truth of my statement.

The following reply to Miss Wood's letter from Mr. Ryan, Joint Honorary Secretary of the Hospitals Association, appeared in the *Evening News* of the 9th inst:—

My attention has but just been drawn to a letter from Miss C. J. Wood, which appeared in your issue of 3rd inst., in which she refers to the Hospitals Association. Into the question of registration of nurses, upon which Miss Wood dilates, I do not propose to enter. A re-discussion of the question would be a mere waste of time, seeing that the most important of the great nurse-training schools in the country declared against registration a year and a half ago. In the opinion of the council of this association, which takes a great interest in nurses, and has done more to improve their position and prospects, by the establishment of the National Pension Fund for Nurses, and by the thorough examination of this registration question, and ascertaining the views of the great nursing schools upon it, than any other agency, an argument upon the subject in the columns of the Press could do no good to nurses, but the reverse. If, however, the British Nurses' Association should persist in the attempt to force into existence a scheme of registration against the declared opinion and wishes of the great nurse-training schools, the time will then have come for these authorities to show cause against the proposal, and to offer thereto all legitimate opposition in their power. In the concluding part of her letter, Miss Wood undertakes to correct what she describes as a misstatement, but which proves to be the diametrical opposite. This being so, the fact that she does not correct it is not to be wondered at. The alleged misstatement is this, that the Hospitals Association originated the scheme of registration. Miss Wood says that she wrote a letter to the association about four years ago, and that as a result of suggestions contained in that letter the association called a conference of hospital matrons, which conference appointed a sub-committee to work out the idea. What was the idea? I have before me at this moment a letter of Miss Wood's, dated as she states, and in it there is no mention whatever of, or allusion to, registration. The idea set forth in this letter is that matrons might be able to utilise each other's experience, and talk over points of common interest, if they met in a conference "under the ægis of the association." This suggestion was made by other influential persons, and the association adopted it, and a conference was held "under the ægis of the association," a sectional committee being formed, which sectional committee framed a set of proposals for the registration of trained nurses. What Miss Wood did was to suggest a conference, as others had done before her; but until the association had called it together, until it had assembled, and until after the sectional committee which it appointed had met, nothing was heard of registration. As a matter of fact, I gather that this question of registration was introduced to the sectional committee by Mrs. Bluett, and not by Miss Wood, for which statement I have the authority of Dr. Bristowe's address at the annual meeting of the association, on May 30, 1888. It will thus be seen that the Hospitals Association first took up this question and thrashed it out, its council, with the chief nurse-training schools, taking the opposite view to that of Miss Wood. She then helped to start the British Nurses' Association, and so to force a hobby on the public and the nurses in the face of the expressed opinion of the authorities, who alone know the facts. Assuming, however, that what Miss Wood states is accurate, such an assumption would not help her argument, as the Hospitals Association first took action, ascertained all the facts, and then, speaking in the name of the authorities interested, declared against the proposals contained in the scheme, which proposals are the same that the British Nurses' Association has been reformulating and refurbishing during the last few months. There is nothing new in these proposals. The whole of them have been before the hospital and nursing world for years, and were negatived as undesirable or impracticable before they were fathered by the British Nurses' Association, as we have evidence to show, and shall proceed to show, when the proper time arrives.

### TROUBLES AT BIRMINGHAM.

Nurse D. writes: I think many of the troubles at Birmingham might be overcome if the authorities would be more charitable in their judgments. It hardly seems fair that the whole bulk of old nurses should be condemned for the behaviour of a few. I can speak from experience that there are nurses of the old staff who have conformed to the very strict rules, and are doing their work cheerfully. No one can tell how hard they must find the restrictions, after living without rule or discipline in the old infirmary. There was much that existed there which astonished one, and it is surprising that so large a town as Birmingham should be in such a benighted condition. It should always be remembered the old nurses have not enjoyed the privilege of training under a strict regime, consequently they cannot so readily see the value of order and discipline. The subject of salaries I have never heard mentioned among them. As to pauper help, now that there are a sufficient number of nurses provided for the work, they no longer wish for it. In the old infirmary it would have been simply impossible for a nurse to attend to the number of cases given her.

### A PLEA FOR NURSES.

Nurse A. W. writes: Will you kindly insert the following in support of Greta's "Plea for Nurses." I am glad she has had the courage to put into words what, I am sure, are the thoughts of many nurses. First, with regard to attending early services. I believe many matrons would more readily grant their nurses late leave to go to a theatre, than allow them simply to give up one hour of their sleep to receive the Blessed Sacrament. Why should this be? and which is most likely to do the nurse harm? Not that I disapprove of theatres and amusements for nurses; on the contrary, I think a certain amount of relaxation after the great strain which the careful fulfilment of a nurse's duties in the sick-room entails, is beneficial, if not absolutely necessary. It is inconsistent for a matron to allow her nurses to stay out late at night after they come off duty, and when they ask leave to go out for an hour before they begin work, for what is a duty as well as a pleasure, to refuse them "out of regard for their health," and yet, if they wish to get up early and go for a walk before breakfast on week days, they are allowed to do so. I trust all matrons will read and think over Greta's letter, and endeavour to assist nurses who wish to obey the Divine command instead of putting a stumbling-block in their way. Let us hope of them the poet's words are true, that "Evil is wrought by want of thought as well as want of heart," and that now their eyes have been opened they will not hinder their nurses from attending early services. With regard to off-duty hours for nurses, I heartily agree with Greta. Workers in other professions know when their hours off duty will be, and can make their arrangements beforehand, and surely nurses, who work harder and have longer hours than most other workers, might be allowed to have a fixed time for going out. By a little care and thought on the matron's part this could be easily arranged, and many sad disappointments avoided.

Beta writes: I have read Greta's opinion, and if it is not "Everybody's Opinion" my opinion is that it ought to be. Memory plays us strange tricks. We are so apt to forget what we do not wish to remember, but I only wish some matrons would endeavour to recall their own feelings when they first entered upon the duties of their profession, and then remember the golden motto "Do as you would be done by," and they as well as the nurses would be the happier for it. Discipline is of the utmost importance, but unless it is tempered by kindness and thoughtfulness it is likely to develop a species of veiled insubordination, and the whole character of the nurse may become gradually and imperceptibly changed, and the "hard expression" that Greta refers to is too often a sign of this change or of the inward battle the nurse has fought in her endeavour to do her duty when she feels that she and others round her are the subjects of arbitrary and unjust treatment. Girls who take to nursing are generally kindhearted and impulsive by nature, with a yearning to do some good in this world before they die. Everything should be done to foster this feeling, for if it is replaced by a feeling of disappointment and resentment, the patients and the whole system of hospital nursing must inevitably suffer. As Greta points out, the young nurse becomes callous and hardened, and so the evil is carried on from generation to generation. In many small hospitals a nurse, when she enters, expects to find in the matron a "mother," and finds a "stepmother."

### Vacancies.

The following vacancies are announced:

**Matrons.**

Fisherton Asylum, Salisbury.

Manchester Eye Hospital, £80.

Nottingham Children's Hospital, £60

Southport Sanatorium, £100.

West Bromwich District Hospital (locum tenens.)

**Sister.**

St. Albans Diocesan Nursing Institution, Hitchin, £35.

**Nurses.**

Aberdeen City Hospital, £26.

Ashford Cottage Hospital.

Bedford Institute (Mental), £26.

Birmingham Fever Hospital.

Bridgwater Infirmary, £22.

Croydon Hospital.

Croydon Union, £20.

Darlington Fever Hospital, £20.

Guildford Hospital, £20.

Hollond Institution, Nice.

Ipswich Hospital, £25.

Jersey Nurses' Institute, £25.

Newlands, Ryde.

Royal Hospital for Diseases of the Chest, £22.

Southampton Institute.

Southport Trained Nurses' Home.

Stratford-on-Avon Hospital, £20.

Stepney Hospital for Children, £25.

St. Leonards Cottage Hospital.

**Midwife.**

Lying-In Home, Shadwell, £40.

**District Nurses.**

Burton-on-Trent, £28.

Clifton, Bristol.

District Home, Bolton, £25.

Maiden Erleigh, Reading.

Stockport Infirmary, £25.

Salford Institution, £25.

Woodsgate, Penbury, £50.

**Probationers.**

Bristol Royal Infirmary.
Southport Infirmary.
West London Hospital.

### Notes and Queries.

To Correspondents.—1. Questions may be written on post-cards. 2. Advertisements in disguise are inadmissible. 3. In answering a query please quote the number. 4. If a private answer is desired, a stamped addressed envelope must be forwarded.

**Queries.**

(41) *Monthly Nursing.*—Where can free training in monthly nursing be obtained? *Nurse J.*

(42) *Situation Wanted.*—Could any of your readers find a situation for a respectable girl of 19, capable of working well, but who imagines she is delicate? She is nice looking, would take low wages, and needs a firm but patient mistress. *M. E. S.*

(43) *Chatelaines.*—Can any nurse tell me where to get a cheap chatelaine with six chains, not fitted? *Surgical Nurse.*

**Answers.**

*Temperance Hospital.*—M. B. M. should apply to the Secretary, London Temperance Hospital, Hampstead-road, N.W.

*Nurse G.*—The address you require is 19, Harley-street.

